# Genome-wide identification of quantitative trait loci for important plant and flower traits in petunia using a high-density linkage map and an interspecific recombinant inbred population derived from *Petunia integrifolia* and *P. axillaris*

**DOI:** 10.1038/s41438-018-0091-5

**Published:** 2019-02-01

**Authors:** Zhe Cao, Yufang Guo, Qian Yang, Yanhong He, Mohammed I. Fetouh, Ryan M. Warner, Zhanao Deng

**Affiliations:** 1 Department of Environmental Horticulture, Gulf Coast Research and Education Center, IFAS, University of Florida, 14625 County Road 672, Wimauma, FL 33598 USA; 2 Department of Horticulture, Michigan State University, East Lansing, MI 48824 USA; 3 Key Laboratory of Horticultural Plant Biology, Ministry of Education, College of Horticulture and Forestry Sciences, Huazhong Agricultural University, 430070 Wuhan, Hubei China; 4 Department of Horticulture, Faculty of Agriculture, Tanta University, Tanta, 31527 Egypt

**Keywords:** Plant genetics, Plant genetics

## Abstract

Petunia is a very important flower in the global floriculture industry and has played a critical role as a model in plant genetic studies. Owing to limited genetic variability in commercial germplasm, development of novel petunia phenotypes and new varieties has become increasingly difficult. To enrich petunia germplasm and facilitate genetic improvement, it is important to explore genetic variation in progenitor species that may contain highly valuable genes/alleles. In this study, an interspecific recombinant inbred population (168 recombinant inbreds) derived from *Petunia integrifolia* × *P. axillaris* were phenotyped for days to anthesis (DTA), flower count (Flower_C), flower diameter (Flower_D), flower length (Flower_L), plant height (Plant_H), plant spread (Plant_S), and plant size (Plant_Z) in 2014 and 2015. Transgressive segregation was observed for all traits in both years. The broad-sense heritability on a 2-year basis varied from 0.38 (Flower_C) to 0.82 (Flower_L). Ten QTL were consistently identified in both years and by two mapping strategies [multiple QTL mapping (MQM) in MapQTL and inclusive composite interval mapping (ICIM) in IciMapping]. Major QTL explained up to 30.2, 35.5, and 47.1% of the total phenotypic variation for Plant_S, Flower_L, and Flower_D, respectively. These findings should be of significant values for introgression of desirable genes from wild petunias into commercial varieties and future genetic improvement of this important flower.

## Petunia: Enhancing commercial cultivars

Uncovering the genetics behind desirable traits in petunia plants could help improve commercial cultivars. While petunia plants are a key feature of the global horticultural industry, the limited genetic variability in commercial plants makes it difficult to improve desired plant traits. Zhanao Deng at the University of Florida, US, and co-workers conducted a genome-wide QTL identification study using a crossbred population combining two petunia  species, *P. integrifolia* and *P. axillaris*. The team phenotyped the petunias in an open-air sub-tropical field rather than in an artificial environment like previous studies. They identified and characterized 17 genetic loci for seven important aesthetic traits, ranging from flower count to plant size. The petunia species had very different genetic backgrounds, likely stemming from their different geographic origins. The two species each can contribute novel genes for enhancing cultivated petunia cultivars.

## Introduction

Cultivated flowers serve a very important role in human life and health, the global economy, and the beautification and protection of the environment. Flower production has become one of the most dynamic and sophisticated sectors of the global horticulture industry. It was estimated that the worldwide production value of cultivated flowers or floricultural production reached 60 billion dollars in 2003^1^. Since then, further growth has occurred in almost every continent. It is projected that the global floriculture market will continue to grow at a compound annual growth rate of 5.4% over the period from 2016 to 2020^2^.

To sustain the global flower production industry, continuous introduction of new cultivars with improved or novel characteristics is essential. Towards this, plant breeders constantly seek to identify novel genes/alleles and combine them into new or improved cultivars. In many crops, including widely produced and used flowers and other ornamental plants^3^, the lack of genetic diversity and lack of novel genes/alleles in the commercial germplasm pool have been limiting plant breeders’ progress in genetic improvement and new cultivar development^4,5^.

Identification and utilization of desirable genes/alleles from wild or progenitor species have been suggested as an effective approach to overcoming this limitation. Enormous efforts have been made in some major agronomic and horticultural crops to characterize wild and ancestor germplasm and identify favourable genes/alleles from the germplasm through phenotyping, genetic mapping, and introgression^6^. On the other hand, wild and ancestor species often perform poorly in horticultural aspects compared to elite germplasm^7^. Wild accessions may carry undesirable genes for the improvement of commercial cultivars^8,9^. When a wild species is crossed with an elite cultivar, the inferior alleles can be simultaneously dragged into cultivars, reducing the plant performance of new cultivars^10^. Numerous rounds of backcrossing are required to reduce or eliminate the inferior donor alleles from elite cultivars, which is a laborious and time-consuming process. Genetic mapping, identification of quantitative trait loci (QTLs), and marker-assisted selection have been used to facilitate the introgression of desirable alleles from wild species to elite cultivars^11^. Over the past two to three decades, several molecular marker systems have been used in such efforts. Genotyping by sequencing (GBS) is a recently developed strategy for large-scale marker discovery^12^. It has been made possible by rapid advances in next-generation sequencing technology. With this strategy, it is possible to sequence hundreds of barcoded samples in a single sequencing lane simultaneously and to reveal single-nucleotide polymorphism (SNP) sites throughout the whole genome. The high output of SNP discovery by GBS has greatly facilitated the construction of high-density, high-resolution genetic linkage maps. GBS has been widely used to construct high-coverage linkage maps and conduct QTL analyses in multiple important agronomic crops^13^.

Garden petunia (*Petunia hybrida*) is a very important flower in the global floriculture production. It is cultivated all over the world and is one of the most important Solanaceae utilized for ornamental purpose^14^. Garden petunia is often among the most popular flowers planted in outdoor gardens in many countries^15^. In the United States, it ranks first in wholesale value among annual bedding plant crops^16^. Cultivated petunia originated from the cross between *Petunia axillaris* and *Petunia integrifolia*^17^. As petunias have been commercially bred with limited germplasm sources for the past 150+ years, the genetic diversity among current commercial cultivars has been low, resulting in high similarities among commercial cultivars and loss of some useful traits^8,18,19^. Several studies have indicated that wild *P. axillaris* and *P. integrifolia* carry traits that may be beneficial to commercial petunia, such as faster development rates^8^, superior freezing tolerance^19^, longer flower longevities^20^, or arthropods resistance^21^. Consequently, interest in introgressing traits from progenitor species to elite petunia cultivars has been strong^21^.

Several genetic linkage maps were developed in petunia, using restriction fragment length polymorphism markers, amplified fragment length polymorphism markers, and simple sequence repeat (SSR) markers^9,17,22–24^. These genetic maps have been used to identify QTL for pollination syndrome traits (length of pistil, stigma, and corolla tube; flower scent; and corolla diameter). A SNP-based linkage map was recently reported in petunia and employed to identify QTL controlling petunia plant development rates (as well as the number of branches and flower buds and days to anthesis (DTA)) under varying temperatures^25^. All reported petunia QTL studies were conducted in the greenhouses using container-grown plants. No or few QTLs have been reported for important aesthetic traits in petunia, including plant size (Plant_Z) and flower count (Flower_C).

In this study, we (1) characterized and phenotyped seven important plant and flower aesthetic traits (DTA, Flower_C, flower diameter (Flower_D), flower length (Flower_L), plant height (Plant_H), plant spread (Plant_S), and Plant_Z) in an open field using a recombinant inbred population derived from a cross between *P. integrifolia* × *P. axillaris* for 2 consecutive years in 2014 and 2015, (2) estimated the heritability for these traits, and (3) identified and located QTL controlling these traits using a high-density SNP bin map developed by the GBS technology.

## Results

### Phenotypic value

**Fig. 1:**
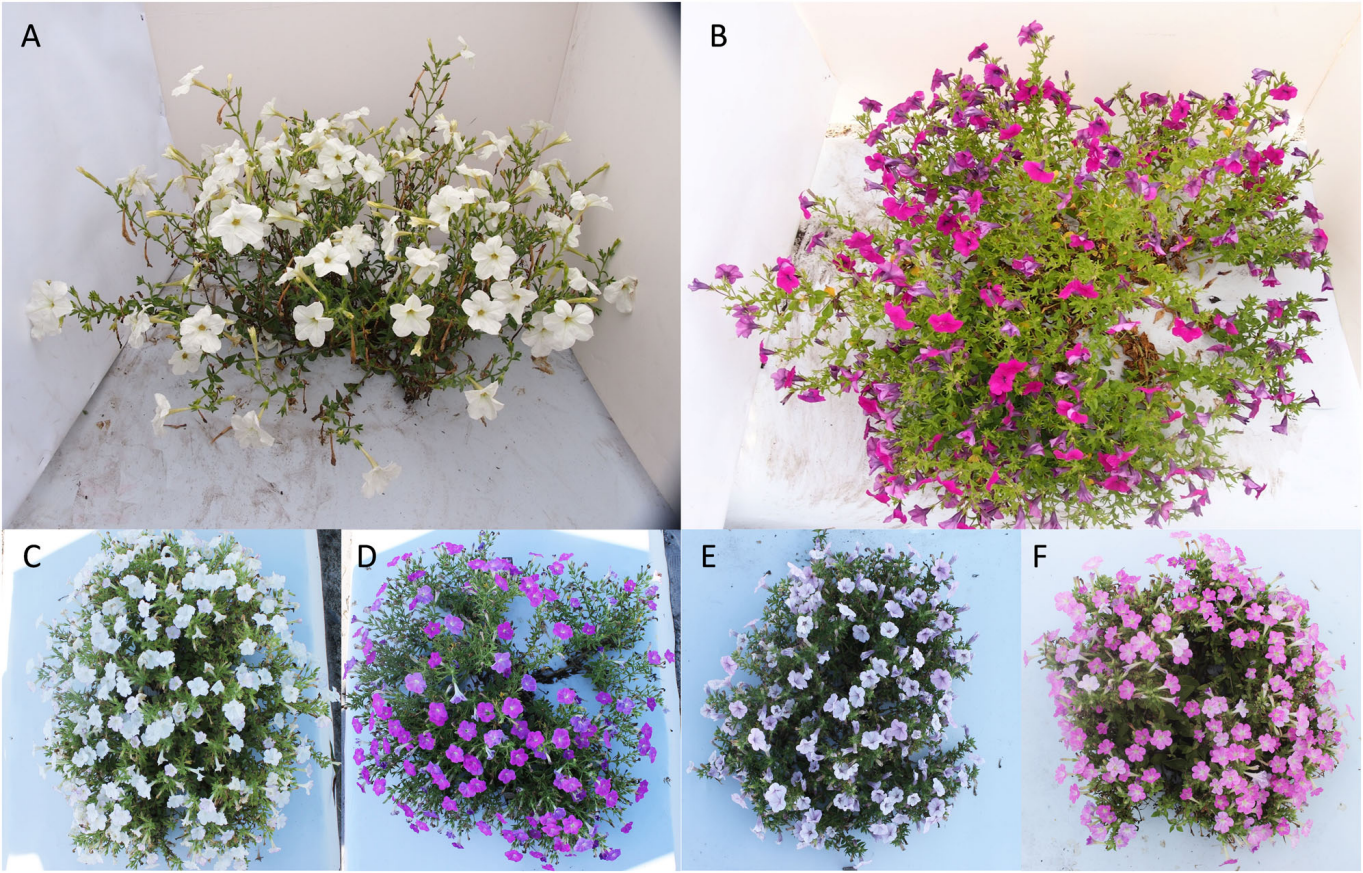
Top view of plants of *P. axillaris*, *P*. *integrifolia*, and four of their RILs grown in the Gulf Coast Research and Education Center (GCREC) experimental farm in Balm, FL, USA (2015). **a** *P. axillaris*. **b** *P. integrifolia*. **c** RIL IA339. **d** RIL IA19. **e** RIL IA100. **f** RIL IA236

**Fig. 2:**
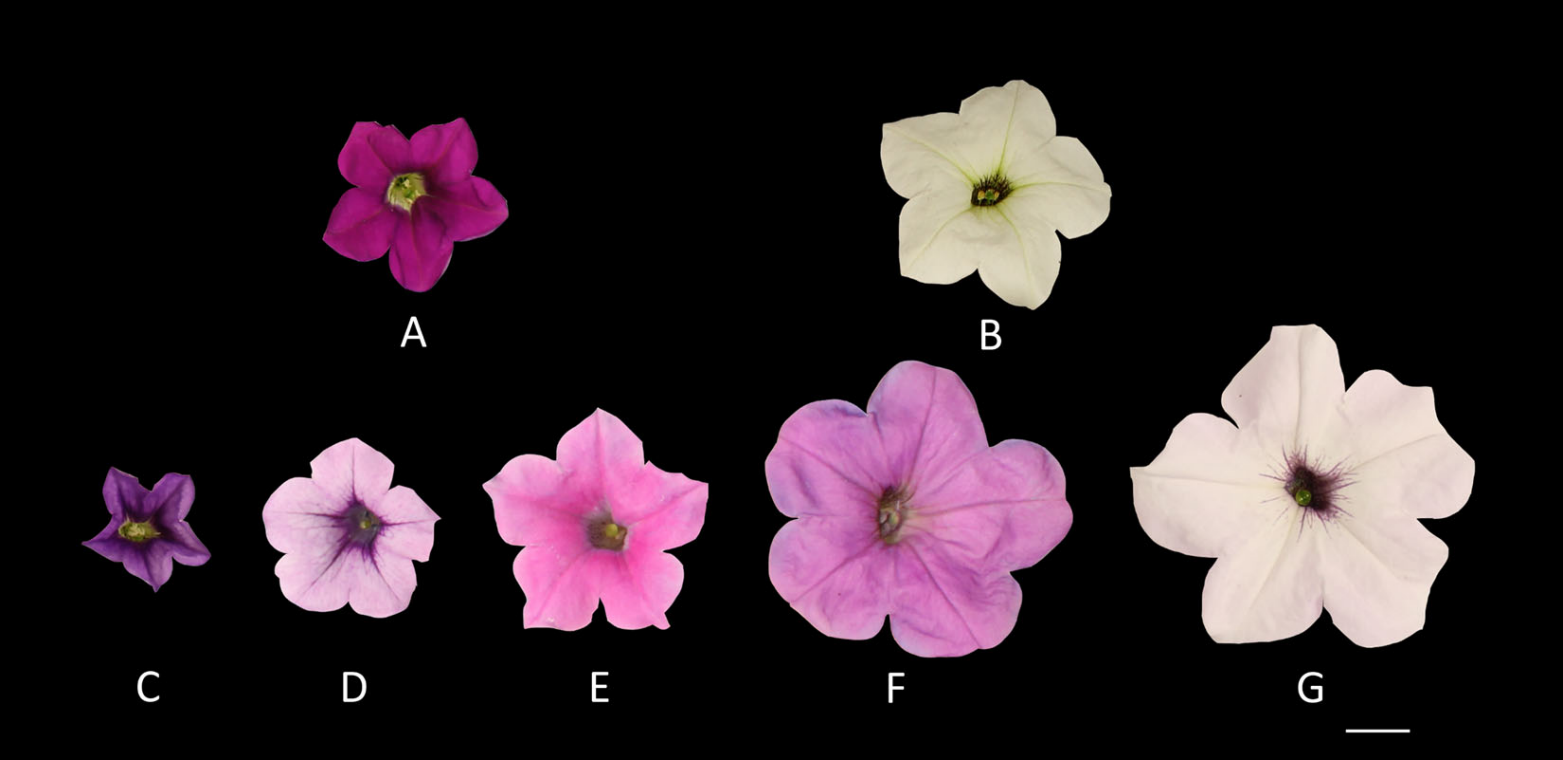
Fully open flowers of *P*. *axillaris*, *P*. *integrifolia*, and five of their RILs showing variations among them in flower size and colour. **a** *P. integrifolia*. **b** *P. axillaris*. **c** IA498. **d** IA73. **e** IA409. **f** IA352. **g** IA403. Scale bar = 1 cm

Phenotypic data including mean value, mid-parents value, and data range for DTA, Flower_C, Flower_D, Flower_L, Plant_H, Plant_S, and Plant_Z of the parents and their recombinant inbred lines (RILs) and the broad-sense heritability (*H*^2^) estimate for each trait based on combined 2-year data are presented in Table 1. Plants of *P*. *axillaris* (Fig. 1) opened first flowers in 75–77 days after seed sowing, each plant produced 211–213 flowers in a period of 7 weeks, the flowers were approximately 5.0 cm long and 5.1 cm wide (Fig. 2), and plants reached an average height of 0.51 m by the end of the growing season. On the other hand, plants of *P. integrifolia* (Fig. 1) first flowered in about 81 days after seed sowing, produced about 779 (in 2014) or 953 flowers (2015) per plant, and reached an average height of 0.32 m by the end of the growing season. Flowers of *P*. *integrifolia* were 2.1–2.5 cm long and 3.4–3.6 cm wide (Fig. 2). *P. axillaris* showed higher values than *P. integrifolia* in Flower_D (45–53% wider), Flower_L (98–141% longer), and Plant_H (53–70% higher), but lower values than *P. integrifolia* in Flower_C (73–78% lower) and DTA (6–9% lower or 5 or 6 days earlier). *P. axillaris* and *P. integrifolia* had similar values for Plant_S and Plant_Z.

**Table 1:** Phenotypic values of *P. axillaris*, *P. integrifolia*, and their recombinant inbred line (RIL) progeny for seven traits

Trait^a^	Year	Phenotypic values						Variance component estimates (%)^e^					Broad-sense heritability^f^
		Parents				Progeny (RILs)							
		*P. axillaris*, mean ± SD	*P. integrifolia*, mean ± SD	*t* Test^b^	Mid-parent value	Mean ± SD	Range (minimum to maximum)	*V*_g_	*V*_e_	*V*_ge_	*V*_b_	*ε*	
DTA (day)	2014	75 ± 2	81 ± 1	*^c^	78	75 ± 11	54–109						
	2015	76 ± 3	82 ± 3	*	79	84 ± 13	58–111	40.18	20.25	25.79	0	13.79	0.40
Flower_C (no.)	2014	211 ± 52	767 ± 86	*	489	289 ± 12	52–819	38.56	2.18	41.3	0	17.91	0.38
	2015	214 ± 26	953 ± 156	*	583	303 ± 158	66–1043						
Flower_D (cm)	2014	5.23 ± 0.05	3.57 ± 0.20	*	4.40	4.06 ± 1.00	1.85–6.28	78.58	2.48	0	1.59	16.94	0.79
	2015	5.12 ± 0.07	3.35 ± 0.09	*	4.24	3.91 ± 0.94	2.33–6.47						
Flower_L (cm)	2014	4.98 ± 0.18	2.51 ± 0.13	*	3.75	3.62 ± 0.77	1.98–5.33	82.42	1.63	1.16	0	14.79	0.82
	2015	5.06 ± 0.14	2.10 ± 0.10	*	3.58	3.60 ± 0.82	1.65–5.67						
Plant_H (m)	2014	0.51 ± 0.04	0.30 ± 0.09	*	0.41	0.37 ± 0.10	0.16–0.63						
	2015	0.52 ± 0.03	0.34 ± 0.05	*	0.43	0.38 ± 0.11	0.14–0.69	69.97	0	4.72	0	26.31	0.70
Plant_S (m)	2014	1.10 ± 0.09	1.05 ± 0.15	ns^d^	1.08	0.67 ± 0.17	0.19–1.20						
	2015	1.09 ± 0.02	1.23 ± 0.13	ns	1.16	0.79 ± 0.26	0.19–1.51	58.53	7.92	14.52	0.9	19.02	0.58
Plant_Z (m3)	2014	0.48 ± 0.05	0.37 ± 0.70 (0.27–0.44)	ns	0.43	0.15 ± 0.11	0.007–0.54						
	2015	0.48 ± 0.06	0.42 ± 0.10 (0.29–0.50)	ns	0.45	0.23 ± 0.19	0.004–0.95	57.98	7.89	10.46	0	23.7	0.58

^a^Trait abbreviations: *DTA* day to anthesis, *Flower_C* flower counts, *Flower_D* flower diameter, *Flower_L* flower length, *Plant_H* plant height, *Plant_S* plant spread, *Plant_Z* plant size. For DTA and FC, the value was rounded off to integers. Two decimal points were kept for Flower_D, Flower_L, Plant_H, Plant_S, and Plant_Z

^b^Four to eight plants for each parental line were included for phenotypic evaluation, and mean separations between *P. integrifolia* and *P. axillaris* were performed by Student’s *t* test

^c^Asterisk (*) represents significance at *P* < 0.05

^d^ns indicates non-significance

^e^*V*_g_, *V*_e_, *V*_ge_, *V*_b_, and *ε* represent the variance components of genotype, environment, genotype × environment, block, and random residue, respectively

^f^Broad-sense heritability equals to the proportion of *V*_g_

**Fig. 3:**
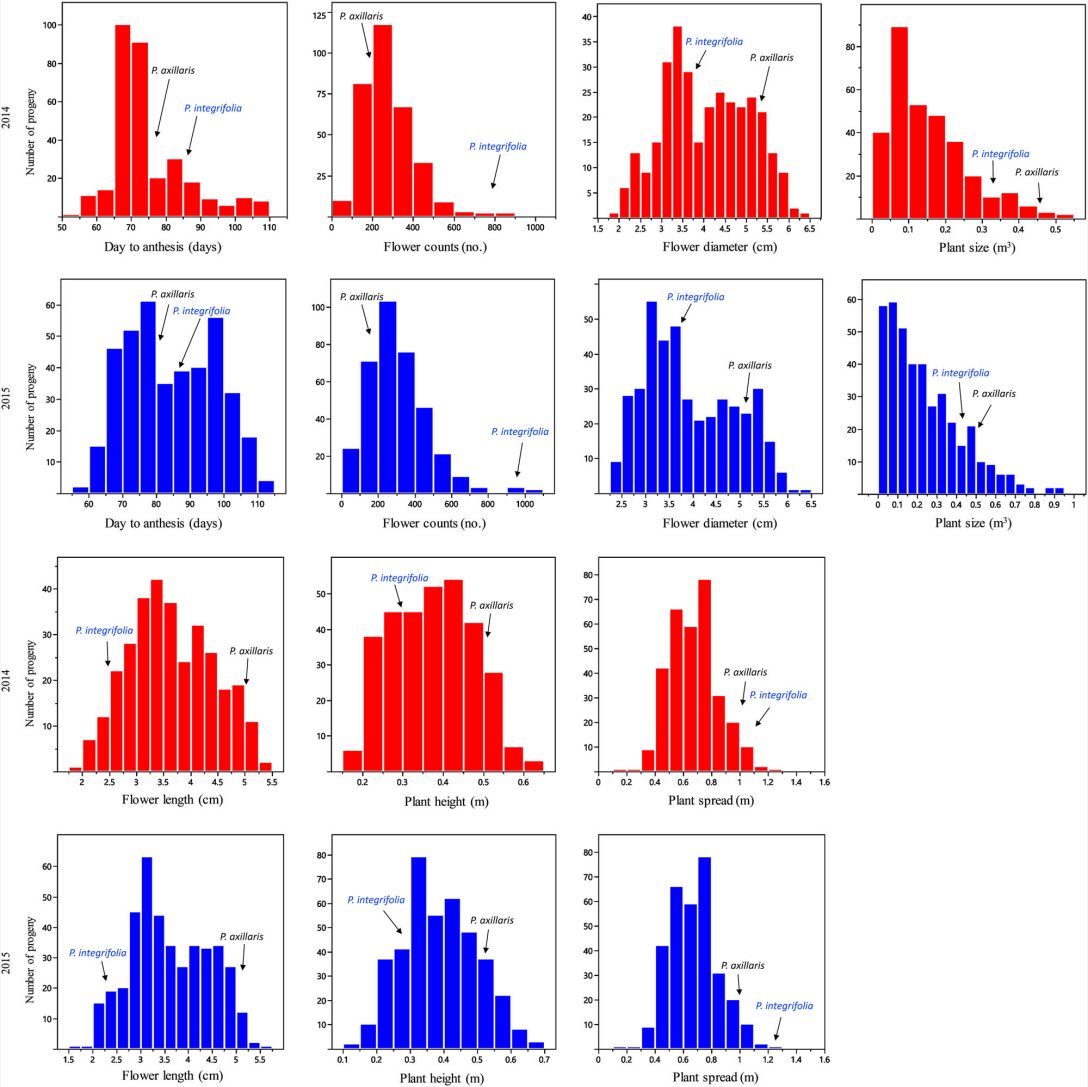
Distribution of progeny of a *P. integrifolia* and *P. axillaris* F_7_ population for seven plant and flower traits in 2014 and 2015. Arrows indicate the mean phenotypic value of *P*. *integrifolia* or *P*. *axillaris* for each trait in respective years

The criteria proposed by Johnson et al.^26^ was used to group the estimated broad-sense heritabilities (*H*^2^) into three categories, low (<0.30), moderate (0.30–0.60), and high (>0.60). According to these criteria, Plant_H (*H*^2^ = 0.70), Flower_D (*H*^2^ = 0.79), and Flower_L (*H*^2^ = 0.82) exhibited high *H*^2^. Moderate *H*^2^ was observed for Flower_C (*H*^2^ = 0.38), DTA (*H*^2^ = 0.40), Plant_S (*H*^2^ = 0.58), and Plant_Z (*H*^2^ = 0.58). Transgressive segregation was observed for all traits studied, including DTA, Flower_C, Flower_D, Flower_L, Plant_H, Plant_S, and Plant_Z (Fig. 3).

Significant correlations were observed between several trait pairs in both 2014 and 2015 (Table 2). Between floral and plant traits, Flower_C and Flower_L were positively correlated with Plant_S and Plant_H, respectively, and their correlation coefficients (*r*) were 0.233 (2014) and 0.312 (2015) for the former and 0.331 (2014) and 0.194 (2015) for the latter. Between floral traits, Flower_D was negatively correlated with DTA (*r* = −0.209 in 2014, and *r* = −0.283 in 2015), but it was positively correlated with Flower_L (*r* = 0.415 in 2014, and *r* = 0.533 in 2015). And between plant traits, Plant_H, Plant_S, and Plant_Z were all correlated with each other; the correlation coefficients were positive between Plant_H and Plant_Z (*r* = 0.612 in 2014, and *r* = 0.570 in 2015) and between Plant_S and Plant_Z (*r* = 0.822 in 2014, and *r* = 0.741 in 2015) but were negative between Plant_H and Plant_S (*r* = −0.201 in 2014, and *r* = −0.167 in 2015). In addition, there were several pairs of traits that were only correlated in either 2014 or 2015, such as DTA and Plant_S (*r* = 0.156 in 2015), Flower_C and Flower_D (*r* = −0.298 in 2014), and Flower_L and Plant_S (*r* = 0.156 in 2014).

**Table 2:** Pearson’s correlation coefficients between traits in a *P. integrifolia* × *P. axillaris* F_7_ RIL population

Trait	DTA	Flower_C	Flower_D	Flower_L	Plant_H	Plant_S
Flower_C	−0.059 (2014)					
	0.033 (2015)					
Flower_D	−0.209** (2014)	−0.298** (2014)				
	−0.283** (2015)	−0.114 (2015)				
Flower_L	0.033 (2014)	−0.117 (2014)	0.415** (2014)			
	0.047 (2015)	−0.038 (2015)	0.533** (2015)			
Plant_H	−0.145 (2014)	0.068 (2014)	0.071 (2014)	0.331** (2014)		
	0.032 (2015)	−0.136 (2015)	0.133 (2015)	0.194** (2015)		
Plant_S	0.121 (2014)	0.233** (2014)	0.089 (2014)	0.156** (2014)	−0.201** (2014)	
	0.156** (2015)	0.312** (2015)	0.103 (2015)	0.0629 (2015)	−0.167** (2015)	
Plant_Z	−0.036 (2014)	−0.071 (2014)	−0.002 (2014)	−0.050 (2014)	0.612** (2014)	0.822** (2014)
	−0.126 (2015)	0.001 (2015)	−0.006 (2015)	−0.029 (2015)	0.570** (2015)	0.741** (2015)

Trait abbreviations: *DTA* (day to anthesis), *Flower_C* (flower counts), *Flower_D* (flower diameter), *Flower_L* (flower length), *Plant_H* (plant height), *Plant_S* (plant spread), *Plant_Z* (plant size)

**Represents significance at *P* < 0.01

### Heterozygosity retention in RILs and at various marker loci

**Fig. 4:**
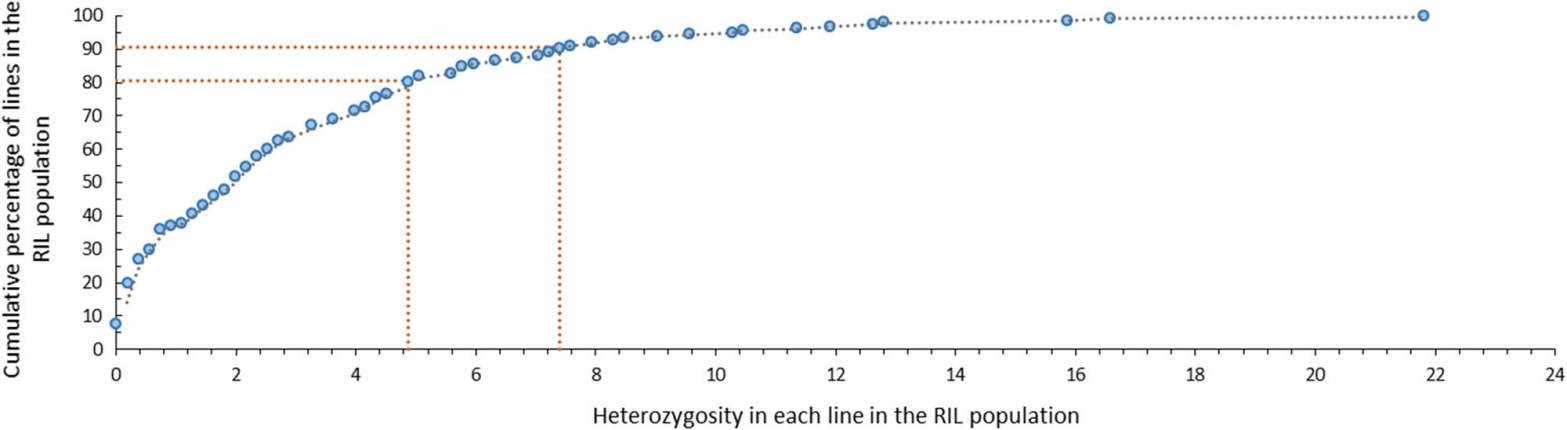
Heterozygosity residues in 168 RILs of an F_7_*P. integrifolia* × *P. axillaris* population. The heterozygosity level of each RIL was calculated by dividing the total number of heterozygous marker loci with the total number of marker loci analysed (482 SNP and 36 SSR marker loci). The *y* axis shows the cumulative percentage of RILs with equal to or lower than a certain percentage of heterozygosity in the *x* axis

We calculated the heterozygosity level in each RIL based on the number of heterozygous marker loci out of the total number of marker loci analysed (518). The heterozygosity level in the 168 RILs ranged from 0 to 21.80% (Fig. 4), averaged to 3.03%, which is much higher than the expected 0.78% (0.5^7^) for an F_7_ RIL population. Overall, ~80% of the RILs in the population had a heterozygosity level below 4.86%, and ~90% of the RILs had heterozygosity level below 7.39%.

**Fig. 5:**
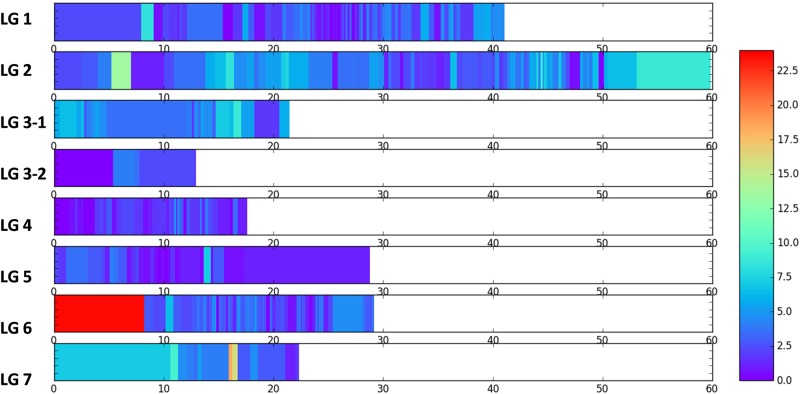
Genome-wide landscape of heterozygosity residues in the petunia genome based on analysis of 482 SNP and 36 SSR marker loci in an F_7_*P. integrifolia* × *P. axillaris* population with 168 RILs. The heat map represents the percentage of heterozygous RIL genotypes out of 168 RILs analysed for each marker locus on each genetic linkage group (LG 1–LG 7)

We also calculated the heterozygosity level for each of the 518 marker loci by dividing the number of heterozygous RILs by the total number of RILs analysed (168). The percentage of heterozygosity per locus ranged from 0% to 23.67%. The average heterozygosity level for the 482 SNP marker loci was 2.70%, while the average heterozygosity for the 36 SSR marker loci was at least one-fold higher, reaching 6.64%. There were 12.74% of the marker loci (66) that had 5–10% heterozygosity, and 1.16% of the marker loci (6) had heterozygosity above 10%. These six loci are all of the SSR type. To show a genome-wide landscape of the heterozygosity residues, the genetic position of each marker locus with its heterozygosity level was plotted on the genetic linkage map (Fig. 5). The retained heterozygosity was not evenly distributed within and among linkage groups (LGs). LG 2, LG 3-1, LG 6, and LG 7 seem to have more regions that retained higher heterozygosity than other LGs. Heterozygosity seemed to be higher in some telomeric regions including the region from 54 to 60 cM on LG 2, the region from 0 to 8 cM on LG 6, and the region from 0 to 12 cM on LG 7.

### QTL detection by multiple QTL mapping (MQM) in MapQTL

**Fig. 6:**
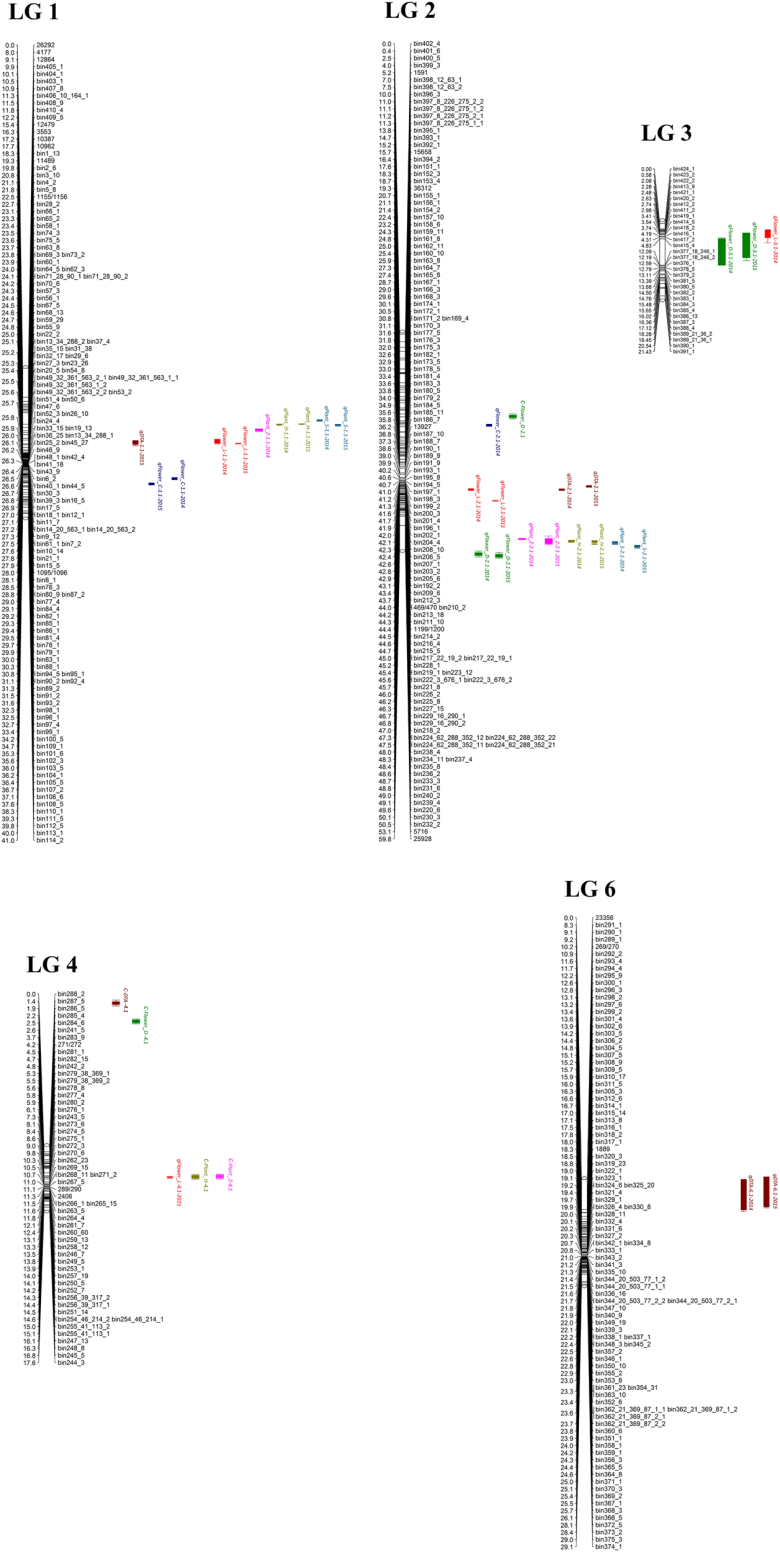
Genetic linkage map and location of QTL for seven petunia traits in a *P. integrifolia* and *P. axillaris* F_7_ population. Markers and their corresponding genetic distances are shown on the right side and left side of linkage groups, respectively. QTL represented by colour bars are on the right of the linkage groups

A total of 17 significant QTL in five LGs were identified for the seven petunia traits (Table 3; Fig. 6).

**Table 3:** Summary statistics of 17 QTL identified by MapQTL for seven plant and flower traits in a *P. integrifolia* × *P. axillaris* F_7_ RIL population in each of 2014 and 2015

Trait^a^	Year	QTL	LOD	LG	Nearest marker	Position	Additive effect^b^	% PVE	LOD threshold
DTA	2014	*qDTA2.1*	4.58	2	Bin209_6	43.372	4.96 (PI)	17.0	2.7
		*qDTA6.1*	3.01	6	Bin291_1	8.252	6.26 (PI)	10.2	
	2015	*qDTA1.1*	3.25	1	Bin4_2	21.144	3.38 (PA)	8.6	2.7
		*qDTA2.1*	6.66	2	Bin204_4	42.136	4.78 (PI)	21.3	
		*qDTA6.1*	3.56	6	Bin291_1	8.252	3.37 (PI)	10.6	
Flower_C	2014	*qFlower_C1.1*	3.75	1	Bin91_2	31.486	52.89 (PA)	14.9	2.7
		*qFlower_C2.1*	4.33	2	Bin174_1	30.13	54.32 (PI)	17.8	
	2015	*qFlower_C1.1*	2.96	1	Bin80_9	28.753	68.01 (PA)	11.4	2.7
Flower_D	2014	*qFlower_D2.1*	15.11	2	Bin232–2	51.458	0.75 (PA)	47.1	2.7
		*qFlower_D3.1*	4.19	3	Bin415_4	8.828	0.37 (PA)	10.3	
	2015	*qFlower_D2.1*	18.08	2	Bin232–2	51.458	0.70 (PA)	44.3	2.7
		*qFlower_D3.1*	5.63	3	Bin415_4	7.828	0.31 (PA)	10.8	
Flower_L	2014	*qFlower_L1.1*	3.80	1	Bin4_2	21.144	0.36 (PA)	11.1	2.7
		*qFlower_L2.1*	8.49	2	Bin212_3	43.744	0.48 (PA)	28.5	
		*qFlower_L3.1*	4.41	3	Bin416_1	4.189	0.88 (PA)	10.4	
	2015	*qFlower_L1.1*	4.27	1	Bin13_34_288_2	25.056	0.25 (PA)	5.5	2.7
		*qFlower_L2.1*	19.35	2	Bin226_2	46.026	0.47 (PA)	35.5	
		*qFlower_L4.1*	4.45	4	Bin274_5	8.388	0.25 (PA)	5.6	
Plant_H	2014	*qPlant_H1.1*	4.26	1	Bin4_2	21.144	0.07 (PA)	16.3	2.7
		*qPlant_H2.1*	5.07	2	Bin232_2	50.458	0.08 (PA)	19.6	
	2015	*qPlant_H1.1*	14.37	1	Bin4_2	21.144	0.08 (PA)	41.1	2.7
		*qPlant_H2.1*	4.54	2	Bin232_2	50.458	0.04 (PA)	10.6	
Plant_S	2014	*qPlant_S1.1*	3.84	1	Bin4_2	21.144	0.16 (PA)	12.9	2.7
		*qPlant_S2.1*	8.12	2	Bin232_2	50.458	0.24 (PA)	30.2	
	2015	*qPlant_S1.1*	3.51	1	1155/1156	22.455	0.13 (PA)	10.8	2.7
		*qPlant_S2.1*	7.10	2	Bin232_2	50.458	0.10 (PA)	23.0	
Plant_Z	2014	*qPlant_Z1.1*	3.71	1	Bin4_2	21.144	0.02 (PA)	13.0	2.8
		*qPlant_Z2.1*	7.74	2	Bin234_11	48.252	0.02 (PA)	29.2	
	2015	*qPlant_Z2.1*	9.56	1	Bin232_2	51.458	0.03 (PA)	29.6	2.7

*PVE* percentage of variation explained

^a^Trait abbreviations: *DTA* (days to anthesis), *Flower_C* (flower counts), *Flower_D* (flower diameter), *Flower_L* (flower length), *Plant_H* (plant height), *Plant_S* (plant spread), *Plant_Z* (plant size)

^b^Additive effect of allele from *P. integrifolia* (PI) or *P. axillaris* (PA)

Days to anthesis (DTA): Three QTLs controlling DTA were *qDTA1.1* (LG 1), *qDTA2.1* (LG 2), and *qDTA6.1* (LG 6). The QTL *qDTA2.1* and *qDTA6.1* were consistently detected in both years, and the percentage of phenotypic variance explained (PVE) by *qDTA2.1* and qDTA6.1 ranged from 10.2% (*qDTA6.1*, in 2014) to 21.3% (*qDTA2.1*, in 2015). The early flowering alleles (beneficial for commercial production) at these loci advanced flowering by 3.37 and 6.26 days, respectively, and they were from *P. axillaris*. The QTL *qDTA1.1* was only detected in 2015, and its PVE was 8.6%. For this locus, the beneficial allele for early flowering (3.38 days) was from *P. integrifolia*.

Flower count (Flower_C): Two QTLs controlling Flower_C were *qFlower_C1.1* (LG 1) and *qFlower_C2.1* (LG 2). The QTL *qFlower_C1.1* was consistently detected in both 2014 (PVE = 14.9%) and 2015 (PVE = 11.4%), while *qFlower_C2.1* (PVE = 17.8%) was identified only in 2014. The *P. axillaris* allele at *qFlower_C1.1* increased the Flower_C by 53–68 per plant, while the *P. integrifolia* allele at *qFlower_C2.1* increased the Flower_C by 54 per plant.

Flower diameter (Flower_D): Two QTLs *qFlower_D2.1* (LG 2) and *qFlower_D3.1* (LG 3) were detected for Flower_D. Both QTL were consistently detected in 2014 and 2015. QTL *qFlower_D2.1* seems to play a more important role, with a PVE of 47.1% in 2014 and 44.3% in 2015. QTL *qFlower_D3.1* had a PVE of 10.3% in 2014 and 10.8% in 2015. Alleles at these loci that contributed to larger Flower_D are from *P. axillaris*, with an additive effect ranging from 0.31 to 0.75 cm per allele.

Flower length (Flower_L): Four QTLs, *qFlower_L1.1* (LG 1), *qFlower_L2.1* (LG 2), *qFlower_L3.1* (LG 3), and *qFlower_L4.1* (LG 4), were detected controlling Flower_L. QTLs *qFlower_L1.1* and *qFlower_L2.1* were detected in both years, and their PVE ranged from 5.5% (*qFlower_L1.1*, in 2015) to 35.5% (*qFlower_L2.1*, in 2015). Two QTLs *qFlower_L3.1* (2014) and *qFlower_L4.1* (2015) were detected only in 1 year, and their PVE were 10.4% and 5.6%, respectively. All beneficial alleles at these loci accounting for longer flowers are from *P. axillaris*, and their additive effect ranged from 0.25 to 0.88 cm in Flower_L per allele.

Plant height (Plant_H): Two QTLs *qPlant_H1.1* and *qPlant_H2.1* were consistently detected in 2 years. The former had a PVE of 16.3% in 2014 and 41.1% in 2015, and the latter displayed a PVE of 19.6% in 2014 and 10.6% in 2015. *P. axillaris* alleles at these loci exhibit additive effects, increasing Plant_H by 0.04 to 0.08 m per allele.

Plant spread (Plant_S): Two putative QTLs *qPlant_S1.1* and *qPlant_S2.1* controlling Plant_S were identified in both years. The locus *qPlant_S1.1* had a smaller PVE of 12.9% in 2014 and 10.8% in 2015, while the QTL *qPlant_S2.1* exhibited a larger PVE of 30.2% in 2014 and 23.0% in 2015. All alleles at these loci for larger Plant_S were from *P. axillaris*, and their additive effects ranged from 0.10 to 0.24 m per allele.

Plant size (Plant_Z): Two putative QTLs identified were *qPlant_Z1.1* (LG 1) and *qPlant_Z2.1* (LG 2). QTL *qPlant_Z2.1* was consistently detected in both years, having a PVE of 29.2% and 29.6% in 2014 and 2015, respectively. QTL *qPlant_Z1.1* was only detected in 2014, and its PVE was 13.0%. And the alleles at these loci for larger Plant_Z were from *P. axillaris*, and their additive effects were between 0.02 to 0.03 m per allele.

### Multi-environmental QTL, QTL × environment interaction (QEI) and epistasis revealed by inclusive composite interval mapping (ICIM) in IciMapping

When two environments (year 2014 and 2015) were jointly considered and the ICIM mapping algorithm was used, a total of 17 QTL were detected for the 7 petunia traits (Table 4). Twelve of these QTLs (70.6%) were also detected by MQM in MapQTL, including 10 environmentally consistent QTLs (*qDTA2.1*, *qDTA6.1*, *qFlower_C1.1*, *qFlower_D2.1*, *qFlower_L1.1*, *qFlower_L2.1*, *qPlant_H1.1*, *qPlant_H2.1*, *qPlant_S2*.1, and *qPlant_Z2.1*) that were detected in both 2014 and 2015 and 2 QTLs (*qDTA6.1* and *qFlower_L4.1*) that were observed in either 2014 or 2015. All QTLs detected by ICIM were located very close to the QTLs detected by MQM, except for two QTLs, *qFLower_L1.1* and *qPlant_H1.1*, which were 9 and 13 cM away from the QTLs by ICIM, respectively.

**Table 4:** Summary table for the position, LOD value, additive effect, and phenotypic variance explained for QTL and QTL × environment interactions detected by IciMapping for seven petunia traits in two environments

Trait^a^	LG	Peak position (cM)	Left CI (cM)	Right CI (cM)	LOD	LOD(A)	PVE	LOD(AbyE)	PVE(AbyE)	AbyE	Detected by MapQTL^b^ (name for new QTL by IciMapping)
DTA	1	20.00	19.75	20.75	4.50	4.04	4.49	0.46	0.09	0.26 (PA)	*qDTA1.1* (2015)
	2	42.50	42.25	42.75	11.91	11.08	13.04	0.83	0.07	0.19 (PI)	*qDTA2.1* (2014 and 2015)
	4	1.00	0	1.75	3.62	3.62	3.98	0	0.15	0.49 (PA)	No (*C-DTA-4.1*)
	6	1.50	0	8.25	3.68	3.57	4.06	0.11	0.01	0.10 (PI)	*qDTA6.1* (2014 and 2015)
Flower_C	1	33.50	31.75	35.25	3.17	3.16	2.92	0.01	0.22	5.68 (PA)	*qFlower_C1.1* (2014 and 2015)
Flower_D	2	29.00	28.25	29.75	10.38	7.34	20.42	3.03	7.38	0.34 (PA)	No (*C-Flower_D-2.1*)
	2	49.50	49.25	49.75	5.94	5.29	10.23	0.65	1.18	0.05 (PA)	*qFlower_D2.1* (2014 and 2015)
	4	3.00	2.75	3.25	5.08	4.6	9.10	0.48	0.71	0.05 (PI)	No (*C-Flower_D-4.1*)
Flower_L	1	30.50	30.25	30.75	5.94	5.14	9.65	0.80	0.23	0.02 (PA)	*qFlower_L1.1* (2014 and 2015)
	2	45.50	45.25	45.75	9.46	4.15	14.62	5.31	6.92	0.28 (PA)	*qFlower_L2.1* (2014 and 2015)
	4	14.00	13.75	14.25	5.08	3.98	12.20	1.10	4.75	0.12 (PA)	*qFlower_L4.1* (2015)
Plant_H	1	34.50	33.75	34.75	7.32	5.98	11.73	1.34	0.93	0	*qPlant_H1.1* (2014 and 2015)
	2	45.50	45.25	45.75	5.51	3.92	12.91	1.58	5.75	0.01 (PA)	*qPlant_H2.1* (2014 and 2015)
	4	13.00	12.75	13.25	5.78	3.57	13.24	2.21	6.86	0.02 (PA)	No (*C-Plant_H-4.1*)
Plant_S	2	49.00	48.75	49.25	8.18	7.73	9.81	0.44	0.04	0	*qPlant_S2.1* (2014 and 2015)
Plant_Z	2	49.00	48.75	49.25	8.06	7.83	23.27	0.23	8.59	0.10 (PA)	*qPlant_Z2.1* (2014 and 2015)
	4	13.00	12.75	13.25	6.60	5.96	12.23	0.64	1.06	0.04 (PA)	No (*C-Plant_Z-4.1*)

*LOD(A)* LOD score for additive effect, *PVE* phenotypic variation explained, *LOD(AbyE)* LOD score for additive × environment effect, *PVE(AbyE)* phenotypic variation explained by additive × environment effect, *AbyE* addictive × environment effect from *P. integrifolia* (PI) or *P. axillaris* (PA) in 2014

^a^Trait abbreviations: *DTA* days to (anthesis), *Flower_C* (flower counts), *Flower_D* (flower diameter), *Flower_L* (flower length), *Plant_H* (plant height), *Plant_S* (plant spread), *Plant_Z* (plant size)

^b^Detected by MapQTL (name for new QTL by IciMapping): Name of QTL detected by MapQTL; if not detected by MapQTL, the name of the QTL newly detected by IciMapping are provided inside the parenthesis

Two QTLs, *C-Flower_D-2.1* and *qFlower_L2.1*, seemed to have significant QEI effects (logarithm of the odds (LOD) > 3.0). The QEI at *C-Flower_D-2.1* explained 7.38% of the phenotypic variation and the QEI at *qFlower_L2.1* accounted for 6.92% of the phenotypic variation. QEI effects increased the Flower_D in 2014 by 0.34 cm and the Flower_L in 2014 by 0.28 cm.

Significant QTL × QTL interactions were observed for two traits, DTA and Plant_Z (Table 5). The interaction between two putative loci, one at 5 cM on LG 2 and the other one at 15 cM on LG 7, explained 8.91% of the DTA phenotypic variation. The additive effect of this digenic interaction reduced the number of DTA by approximately 2 days. The interaction between another two putative loci at 50 cM on LG 2 and 10 cM on LG 6 explained 5.88% of the Plant_Z phenotypic variation. The additive effect of the interaction between these loci increased the Plant_Z by 0.09 m. The epistasis × environment effects were not significant for DTA and Plant_Z, as their LOD(AAbyE) score were only 0.02 and 1.35, respectively.

**Table 5:** Epistatic QTL detected for two petunia traits in two environments

Traits	Linkage group	Position 1 (cM)	Linkage group	Position 2 (cM)	LOD	PVE	AddbyAdd	LOD(AAbyE)
DTA	2	5.00	7	15.00	5.34	8.91	−2.16	0.02
PZ	2	50.00	6	10.00	5.00	5.88	0.10	1.35

*PVE* phenotypic variation explained, *AddbyAdd* epistatic effect between two QTL, *LOD(AddbyE)* the LOD score for epistasis × environment interaction

^a^Trait abbreviations: *DTA* (days to anthesis), *Plant_Z* (plant size)

## Discussion

Significant phenotypic variation was observed for all seven traits (DTA, Flower_C, Flower_D, Flower_L, Plant_H, Plant_S, and Plant_Z) in the F_7_ RIL population derived from the cross between *P. integrifolia* and *P. axillaris* (Table 1 and Fig. 3). All traits exhibited certain degrees of transgressive segregation in the RIL population. These results suggest that *P. axillaris* and *P. integrifolia* possess a very different genetic background. This is probably because *P. axillaris* and *P. integrifolia* evolved from two different ecogeographic isolations^27^ and have developed their own pollination syndromes, which have restricted natural gene flows between the two species^28^. Similar transgressive segregation was previously reported for Flower_D, Flower_L, and DTA in *P. integrifolia* × *P. axillaris* F_2_ populations^8,9^.

Traits with higher *H*^2^ across different growing environments and/or seasons can be reliably selected in breeding. In this study, Plant_H (*H*^2^ = 0.70), Flower_D (*H*^2^ = 0.79), and Flower_L (*H*^2^ = 0.82) were found to have high heritability. Similar results were reported in previous petunia studies^8,9,29^. Four traits, including Flower_C (*H*^2^ = 0.38), DTA (*H*^2^ = 0.40), Plant_S (*H*^2^ = 0.58), and Plant_Z (*H*^2^ = 0.58), have moderate *H*^2^, suggesting that these traits are more sensitive to environmental changes. Moderate *H*^2^ was observed for DTA in previous studies^8,9^. Similar *H*^2^ estimates were also reported for Plant_S, Plant_Z, and Flower_C in a *P. axillaris* × *Petunia exserta* RIL population^30^.

In both 2014 and 2015, positive correlation was observed between Plant_S and Flower_C (*r* = 0.233 in 2014, and *r* = 0.312 in 2015) and between Plant_H and Flower_L (*r* = 0.331 in 2014, and *r* = 0.194 in 2015) (Table 2). These correlations suggest that plants with wider breadths are likely to produce more flowers, and taller plants tend to produce longer flowers. Similar results had been reported in an F_2_ population of *P. integrifolia* × *P. axillaris*^8^ and an F_7_ population of *P. axillaris* × *P. exserta*^30^. The negative correlations between Flower_D and DTA (*r* = −0.209 in 2014, and *r* = −0.283 in 2015*)* suggested that selecting large flowers (in diameter) could compromise Flower_C. The moderate correlation coefficient (*r* = 0.415 in 2014, and *r* = 0.533 in 2015) between Flower_D and Flower_L indicated that flowers with larger corolla sizes tended to be longer. While the negative correlation between Plant_H and Plant_S (*r* = − 0.201 in 2014, and *r* = −0.167 in 2015) indicated that selecting higher wider plants may sacrifice Plant_H, other pairs of traits only exhibited significant correlation in either of 2014 and 2015. This might be due to phenotypic variability between years, especially for some low heritable traits, such as DTA and Flower_C.

Theoretically the average heterozygosity in an F_7_ inbred population should be 0.78%, as the heterozygosity reduces by half for each cycle of inbreeding. In this study, we observed a much higher level of heterozygosity (3.03%) in the F_7_ interspecific petunia population (Figs. 4 and 5). Higher levels of heterozygosity were also observed in several telomeric regions of the LGs. This phenomenon was observed in several previous studies^31,32^. The biological meaning for retaining these heterozygous segments in petunia remains to be understood. One hypothesis might be that these segments are important to petunia growth and development, and when they become homozygous or fixed, petunia plants may have reduced fitness.

To identify consistent, useful QTL in petunia, we phenotyped a large number of RILs over two growing seasons and replicated each RIL several times in each growing season. By using MQM in MapQTL, a total of 17 putative QTL controlling seven important petunia plant and flower traits were observed. Twelve of these QTL were also confirmed by ICIM in the IciMapping software. Ten QTL were detected in both 2014 and 2015 and by both mapping strategies (MQM and ICIM). Some of these consistent QTL explained large proportions of phenotypic variance [*qPlant_Z2.1*, *PVE* = 29.2% (2014) and 29.6% (2015) in MQM, and 23.3% in ICIM; *qFlower_L2.1*, *PVE* = 28.5% (2014) and 35.5% (2015) in MQM, and 14.6% in ICIM; *qFlower_D2.1*, *PVE* = 44.3% (2015) and 47.1% (2014) in MQM, and 20.4% in ICIM].

Previously, Guo et al.^25^ used the same population, linkage map, and mapping algorithm (MQM) as we did in this study to identify and locate QTL for the traits Flower_D and DTA. The distinct difference between Guo et al.^25^ and this study was that the population was previously phenotyped in an artificial growing environment (greenhouses with precise temperature and light control) in a temperate climate while the population was phenotyped in open fields in a subtropical climate in this study. Guo et al.^25^ detected the QTL *FD2.1* at 30.49 cM to 31.78 cM on LG 2 for Flower_D. A QTL (*C_Flower_D-2.1*) was detected for petunia Flower_D in the present study by using the ICIM strategy. *C_Flower_D-2.1* is located at 28.25 cM to 29.75 cM on LG 2, very close to *FD2.1*. We projected the confident intervals of *FD2.1* and *C_Flower_D-2.1* to the *P. axillaris* genome and found one common scaffold Peaxi162Scf0007 (Supplemental Table S1). This scaffold contained 149 genes (Supplemental Table S2); two of the genes (*Peaxi162Scf0007g00237* and *Peaxi162Scf01617g*) are predicted to be involved in plant auxin synthesis. It is known that auxins play an important role in regulating plant flower initiation and organ growth^33,34^ (Supplemental Table S3); these two genes may be considered as candidate genes for petunia Flower_D regulation. Two other genes (*Peaxi162Scf0007g02848* and *Peaxi162Scf0007g02859*, coding for an F-box protein and the developmental-regulator-ULTRAPETALA, respectively) (Supplemental Table S3) are predicted to be involved in floral meristem initiation or expansion in petunia, tomato, or *Arabidopsis*^35,36^. These two genes may be considered as candidate genes for petunia Flower_D regulation as well.

One of the Flower_L QTL, *qFlower_L2.1*, was located at 43.74 cM (LG 2) when the 2014 phenotyping data were analysed but shifted to 46.03 cM (LG 2) when the 2015 phenotyping data were analysed. Significant QEI (LOD = 5.31) was also observed for this QTL. We suspect that this QEI might have played some role in the QTL peak position shift between years or environments. Comparing QTL from Guo et al.^25^ with those from the present study led to the recognition that two pairs of QTL for DTA, *qDTA1.1* and *DTA1.1*^25^ and *qDTA6.1* and *DTA6.1*^25^, were mapped onto the same LGs, but in different regions in the LGs (around 21 cM for *qDTA1.1* and around 31 cM for *DTA1.1*; around 8 cM for *qDTA6.1* and around 19 cM for *DTA6.1*). It remains to be determined whether these QTL represent different loci or have shifted positions between studies. The trait DTA had low *H*^2^ and was prone to be influenced by environment conditions, and the two studies were completed in very different growing environments (greenhouses with very good temperature and light control in a temperate climate^25^ vs. open fields in a subtropical climate). These factors might contribute to change of QTL peak position between studies.

Results from this study showed that *P. integrifolia* possesses beneficial alleles that can increase Flower_C, while *P. axillaris* carries favourable alleles for high Flower_C, increased Flower_L and Flower_D, and larger Plant_Z. These alleles are of significant value for introgression into commercial petunia cultivars. Gene introgression from wild species to commercial cultivars has been seldom reported in ornamental plants, but it has been practised frequently in tomato^37^, rice^38^, and other crop species. It is estimated that 30–50% of major QTL from wild species or progenitor species could be beneficial for commercial breeding^35^. The major and environmentally stable QTL identified in this study can be very useful for further genetic improvement of petunia. The QTL information could facilitate the development of trait-associated molecular markers and accelerate marker-assisted transfer of favourable QTL from wild species to commercial cultivars while minimize dragging of chromosomal fragments containing deleterious genes (linkage drag) into commercial germplasm.

The three QTL-rich segments in LG 1, LG 2, and LG 3 (Fig. 6) indicate the presence of linkage among different alleles or pleiotropic alleles that control two or multiple traits. This phenomenon has been observed in other plant species, such as *Brassica napus*^39^, sorghum^40^, and sweet cherry^41^. In an F_7_ *P. axillaris* × *P. exserta* population, the presence of QTL-rich chromosomal regions were also observed LG 1, LG 2, and LG 4^42^. These results indicated that QTL-rich chromosomal regions may be common in petunia and may have additional value for petunia breeding.

## Materials and methods

### Plant material

The RIL mapping population consisted of 168 individuals and was developed by crossing *P. integrifolia* (PI 28546, from the USDA Ornamental Plant Germplasm Center, Columbus, OH) and *P. axillaris* (PI 28546; USDA Ornamental Plant Germplasm Center) and selfing their progeny for seven generations (F_7_) following a single seed descent procedure. This mapping population was previously described by Guo et al.^25^. *P. axillaris* exhibits an apical dominance growth habit, long internodes, long floral tubes, and large floral limbs. In contrast, *P. integrifolia* has a creeping growth habit, short internodes, short floral tubes, and small floral limbs.

### Replicated field experiments for phenotyping

In early January of each year (2014 and 2015), seeds of the RILs and their parents were sowed into 20-row germination trays (27.94 cm in width × 30.48 cm in length). Seeds were germinated in a growth chamber at the University of Florida’s Gulf Research and Education Center (UF/GCREC) at a constant temperature (27 °C) and under an ambient light intensity of 150 μmol m^−2^ s^−1^. Two weeks later, the germination trays with young seedlings were transferred to a greenhouse where the air temperature was maintained between 25 °C and 30 °C. After 12 days, six seedlings were individually transplanted to 72-cell trays (66.04 cm in length × 33.02 cm in width) filled with a commercial soilless substrate (Fafard^®^ 3B; Conrad Fafard, Agawam, MA, USA). The seedlings were grown in the same greenhouse until they were ready to be transplanted to ground beds. Seedlings were fertilized twice weekly using a commercial water-soluble fertilizer containing 15% (w/w) total nitrogen, 5% phosphate (P_2_O_5_), and 15% potassium (K_2_O) (Peters^®^ Excel; Everris NA, Dublin, OH, USA). Two weeks later, all seedlings were moved to a shade house with 30% shade and kept there for 1 week to acclimate the seedlings to the outdoor environment. After the acclimation, four seedlings per RIL and parent were transplanted to mulched, raised ground beds in the UF/GCREC experimental farm (central Florida; N 27^o^ 45”, S 82^o^ 13”). The ground beds were fumigated with Pic-Clor 60 (60% chloropicrin and 40% 1,3-dichloropropene) at 45 kg per 1000 m^2^ 1 month prior to transplanting. Transplanted petunia plants were irrigated with a drip irrigation system 30 min a day. Each plant received 8 g of controlled-release fertilizer Osmocote® (The Scotts Miracle-Gro Company, Marysville, OH, USA). During the petunia growing season (late February to mid-June), the daily average air temperature ranged from 11 °C to 28 °C in 2014 and from 6 °C to 28 °C in 2015. The total precipitation during the growing season was 42.39 cm in 2014 and 46.30 cm in 2015. The experiments in both years followed a randomized complete block design, with four replicates and one RIL plant per experimental unit.

### Collecting phenotype data

In each year, the large RIL population as well as their parents was phenotyped for seven plant and floral traits of the most significance to the use of petunia as a bedding and garden plant, including DTA, Flower_C, Flower_D, Flower_L, Plant_H, Plant_S, and Plant_Z. DTA were calculated as follows: DTA (days) = date of first flower anthesis − date of seed sowing. When approximate 50% of progeny came into flowering, all flowers on each plant were counted weekly for 7 weeks to obtain Flower_C data. Three fully opened flowers per RIL and parent were randomly selected to collect data for Flower_D and Flower_L. Flower_D was measured from one side of the flower’s petal to the opposite side; Flower_L was measured from the base of the flower’s calyx to the top of the flower’s corolla. Plant_H and Plant_S were recorded near the end of the growing season (early June to mid-June). Plant maximal spreads were measured along the longest axis between two opposite margins of the plant. While plant minimal spread was taken along a straight line between two plant margins that were perpendicular to the maximal Plant_S. Two directional Plant_S (plant maximal and minimal spread) were collected and then averaged to represent Plant_S. Plant_H was measured from the bed surface to the highest point of the plant. The value of Plant_Z was determined by Plant_H, plant maximal spread, and plant minimal spread and calculated using the formula: Plant_Z (m^3^) = [π × (plant maximal spread ÷ 2) × (plant minimal spread ÷ 2) × Plant_H].

### Statistical analysis

The statistical software JMP Pro 10.0.2 (SAS institute Inc., Cary, NC, USA) was used to calculate progeny distribution for each trait studied and Pearson’s correlation coefficients and to estimate the broad-sense heritability for each trait. All board-sense heritability (*H*^*2*^) estimates were calculated using the following statistical model: *y*_*ijk*_ = *µ* + G_*i*_ + *E*_*j*_ + *G*_*i*_ × *E*_*j*_ + *B*_*h(j)*_ + *ε*_*ijk*_, where *y*_*ijk*_ represents the measured phenotypic value of the studied trait for individual plant_*ijk*_, *µ* the population mean value for the specific trait, *G*_*i*_ the genetic effect, *E*_*j*_ the environment effect, *G*_*i*_ × *E*_*j*_ the effect of interactions between genotype and environment, *B*_*h(j)*_ the block effect, and *ε*_*ijk*_ the random error. All components (G_*i*_, *E*_*j*_, *G*_*i*_ × *E*_*j*_, *B*_*h(j)*_, and *ε*_*ijk*_) in this model were treated as random effects.

### Calculation of heterozygosity in RILs and at marker loci in LGs

The heterozygosity level of each RIL and at each marker locus was calculated using the marker genotyping data described by Guo et al.^25^. The genotyping data consist of data from 482 SNP and 36 SSR markers. Molecular marker genotypes were categorized into either being heterozygous or homozygous. The level of heterozygosity (%) in each RIL was calculated by dividing the total number of heterozygous marker loci in each RIL by the total number of marker loci analysed. The level of heterozygosity at each marker locus was obtained by dividing the total number of heterozygous RILs by the total number of RILs analysed. The resulting data were plotted in the software Matplotlib^43^ to show a genome-wide landscape of heterozygosity retention with and among LGs.

### QTL identification and analysis

The genetic linkage map described by Guo et al.^25^ was used for QTL identification and localization in this study. The genetic map contained 518 bins (482 SNPs and 36 SSRs) spanning a total genetic distance of 220.2 cM across petunia’s seven chromosomes. Molecular markers in this genetic map could be located to 620 scaffolds, 0.74% of the total number of scaffolds in the assembled *P*. *axillaris* genome (https://solgenomics.net/organism/Petunia_axillaris/genome)^42^. Nevertheless, these 620 scaffolds contain 747,650 kb of nucleotides, which is approximately 53.4% of the *P*. *axillaris* genome (1.4 Gb). The software MapQTL 6.0^44^ was employed for QTL analysis. Putative QTL regions were first determined by interval mapping and the resulting highest scored markers were then highlighted and labelled. These stamped markers were subsequently treated as cofactors and run in MQM. The LOD thresholds for putative QTL were determined by permutation tests (1000 times per run) with the significant threshold at 95th percentile of LOD scores. Only QTL with a LOD score more than the LOD threshold value were declared and retained in the analysis and reported here. To verify the QTL detected in MapQTL and to estimate the QEI and QTL × QTL (epistasis) effects, the ICIM model software IciMapping 4.1^45^ was used; the LOD threshold value 3.0 was used to declare significant QTL and QEI, and the LOD cutoff of 5.0 was used to declare the presence of epistasis.

## Electronic supplementary material

Supplemental Table S1

Supplemental Table S2

Supplemental Table S3
